# Ordered arrangement of F4TCNQ anions in three-dimensionally oriented P3HT thin films

**DOI:** 10.1038/s41598-020-77022-0

**Published:** 2020-11-18

**Authors:** Shuichi Nagamatsu, Shyam S. Pandey

**Affiliations:** 1 Department of Physics and Information Technology, Kyushu Institute of Technology, 680-4 Kawazu, Iizuka, Fukuoka, 820-8502 Japan; 2 Graduate School of Life Science and Systems Engineering, Kyushu Institute of Technology, 2-4 Hibikino, Wakamatsu-ku, Kitakyushu, 808-0196 Japan

**Keywords:** Materials science, Materials for devices, Soft materials

## Abstract

An ordered arrangement of electron-accepting molecular dopant, 2,3,5,6-tetrafluoro-7,7,8,8-tetracyanoquinodimethane (F4TCNQ), in three-dimensionally (3D) oriented poly(3-hexylthiophene) (P3HT) film was clarified. The 3D oriented P3HT thin films prepared by the friction-transfer technique were doped with F4TCNQ by dipping into an acetonitrile solution. The presence of F4TCNQ anions in the 3D oriented P3HT thin films was investigated by polarized ultraviolet/visible/near-infrared absorption spectroscopy, grazing incidence X-ray diffractometry, polarized Fourier transform infrared spectroscopy (FT-IR), and infrared *p*-polarized multiple-angle incidence resolution spectroscopy (pMAIRS). The F4TCNQ-doped 3D oriented P3HT films showed anisotropic properties in all characterizations. In particular, the anisotropic molecular vibrations from polarized FT-IR and pMAIRS have clearly revealed orientations of polymeric chains and molecular dopant molecules. Considering the results from several independent techniques indicated that F4TCNQ anions in the 3D oriented P3HT were orderly arranged in a 3D manner with respect to the 3D oriented P3HT such that their molecular long-axis parallel to the P3HT backbone, with in-plane molecular orientation. Additionally, the direction of the optical transition moment of the F4TCNQ anion was found to be parallel to the molecular short-axis.

## Introduction

Conjugated polymers (CPs) have shown their potentials as active materials for organic semiconducting devices, such as organic light-emitting diodes (OLEDs), organic thin-film transistors (OTFTs) and organic photovoltaics (OPVs) owing to their ability to easily tune the material properties and for the low-cost manufacturing of organic electronic devices by solution processing^1–5^. Charge-transfer (CT) doping of CPs by electron acceptors is widely used for the development of efficient organic semiconducting devices^6–9^. Acceptor doping of thiophene-based CPs with 2,3,5,6-tetrafluoro-7,7,8,8-tetracyanoquinodimethane (F4TCNQ) has attracted significant attention owing to the deeper lowest unoccupied molecular orbital (LUMO) levels of this acceptor, which is adequately deep to oxidize the highest occupied molecular orbital (HOMO) levels of several CPs^10–13^. Stable doping of regioregular poly(3-hexylthiophene) (P3HT) by F4TCNQ can be achieved by the blended solution method^14–19^, and by the sequential processing method^20–28^. A significant increase in the electrical conductivity of up to five orders of magnitude was reported for F4TCNQ-doped P3HT. From literature, solution phase doping from blended solution and step-wise doping have been used; however, stepwise film casting followed by doping was proposed by Jacobs *et al*. to be superior because of the attainment of higher conductivity even at lower doping levels, and its compatibility with roll-to-roll fabrication processes^22^. This was explained by the fact that sequentially doped films bear several enhanced tie molecules between the crystalline domains formed before doping. In the blended system, the doped particles repel each other owing to the negative surface dipole formation aided by the surrounding polymeric crystals by dopant anions that form discontinuous polymer domains during thin film fabrication. This indicates an improvement in the charge transport is owing to the synergistic effect of the development of oriented thin films followed by doping leading to facile access of dopant molecules with the polymeric main chain. These doped CPs are utilized in organic electronic devices as carrier transport or injection layer. Therefore, there is an urgent requirement to investigate the doping mechanism of P3HT by F4TCNQ, particularly focusing on the implications of the molecular arrangement in F4TCNQ-doped P3HT thin films^13–15^. However, in the combined F4TCNQ-doped P3HT, it is difficult to investigate the molecular arrangement owing to the randomly oriented P3HT domains and random distribution of F4TCNQ dopant molecules in the thin film preparation and doping processes. An integrated theoretical and experimental approach based on computations and X-ray diffraction (XRD) with inelastic neutron scattering was used to investigate the molecular arrangement of F4TCNQ doped P3HT prepared by solution phase doping from a mixture of polymer and dopant^9,14,15^. From the study, it was concluded that dopant molecules are present between the π–π stacking of the main chain in a face-to-face manner. The arrangement may differ in the same polymeric system with pre-existing ordered domains.

Recently, several studies have been conducted to investigate the arrangement of dopant molecules in uniaxially oriented polythiophene derivatives such as P3HT and poly(2,5-bis(3-alkylthiophene-2-yl)thieno[3,2-*b*]thiophene) (PBTTT), wherein the doping was performed after the orientation of the CPs under investigation^12,13,24–26^. This post-doping of the oriented thin films is advantageous over blend systems to investigate the molecular arrangement of dopant molecules explicitly. Based on the optical absorption and XRD results, it was proposed that F4TCNQ molecules are incorporated into the side-chains region of oriented PBTTT, and the F4TCNQ long-axis is perpendicularly oriented to the polymer backbones^13,25,26^. This hypothesis was based on the findings that direction of the optical transition moment of the TCNQ anion was parallel to the molecular long-axis by the liquid crystalline matrix induced orientation of the dicyclohexyl-18-crown-6 complex of potassium TCNQ in 1976^29^. We emphasize that, in this system, the orientation of the molecule was significantly small and may not be true for highly oriented systems. We propose that complex systems should not be generalized, and an in-depth understanding of the direction of the optical transition moment of TCNQ anion depending on the CP-TCNQ anion system in terms of the nature and extent of alignment under consideration is required. Optical absorption studies are significantly useful for the investigation of molecular orientation provided that the direction of the optical transition moment is known.

Several methods using sheer forces, such stretching and mechanical rubbing, were reported for obtaining the uniaxial orientation in CP films^24,25,30^. Herein, we proposed unique methods such as friction-transfer^31–36^, and floating-film transfer^37–44^, to obtain highly oriented and crystalline thin films of CPs. Simultaneously, these methods were used for the alignment of CPs such as polythiophene, poly(p-phenylenevinylene), polyfluorene and their derivatives to demonstrate their anisotropic optoelectronic properties. Moreover, the analysis of friction-transferred films provided in-depth information regarding the crystal structure of CPs and indicated that CP molecules were arranged not only uniaxially but also three-dimensionally (3D)^31,33,34,36^. Tanigaki *et al.* succeeded in the post-doping of dye molecules in friction-transferred CP films by the vapor transportation method and emphasized that doped dye molecules were aligned with respect to the alignment of polymer backbones. Furthermore, they observed polarized white emission from a friction-transferred blue emissive polyfluorene film doped with orange emissive oligothiophene^45,46^.

**Figure 1:**
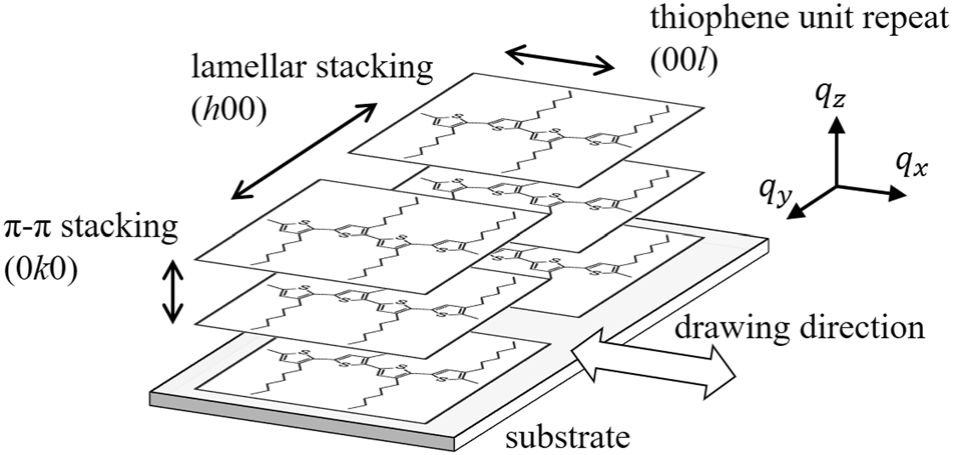
Schematic of the molecular arrangement in the friction-transferred P3HT film.

Macromolecules in the friction-transferred P3HT film have 3D molecular arrangement, with the backbone along the drawing direction of the friction-transfer, while alkyl side-chains arrange themselves in the film plane in the face-on orientation, as schematically shown in Fig. 1. Therefore, in such doped systems, F4TCNQ dopant molecules are expected to be arranged in 3D manner with respect to the 3D oriented friction-transferred P3HT film. The F4TCNQ post-doping of P3HT films was performed by a simple dipping method^27,28^. In this study, we performed an in-depth investigation to determine the arrangement of the F4TCNQ and P3HT molecules in the F4TCNQ-post-doped oriented P3HT films by polarized ultraviolet–visible-near infrared (UV–vis-NIR) absorption spectroscopy, grazing incidence X-ray diffraction (GIXD) analyses, polarized Fourier transform infrared (FT-IR) spectroscopy and infrared p-polarized multiple-angle incidence resolution spectroscopy (pMAIRS)^47–50^. By focusing on the C≡N stretching vibrations in F4TCNQ, FT-IR and pMAIRS results provided significant information for the direct investigation of TCNQ molecular orientation. From these analyses, post-doped F4TCNQ anions were arranged in 3D manner in the side-chain region of P3HT, with F4TCNQ molecular long-axis parallel to the P3HT backbones, while F4TCNQ molecules lie in the film plane.

## Results and discussion

### Polarized UV–Vis-NIR absorption spectroscopy

**Figure 2:**
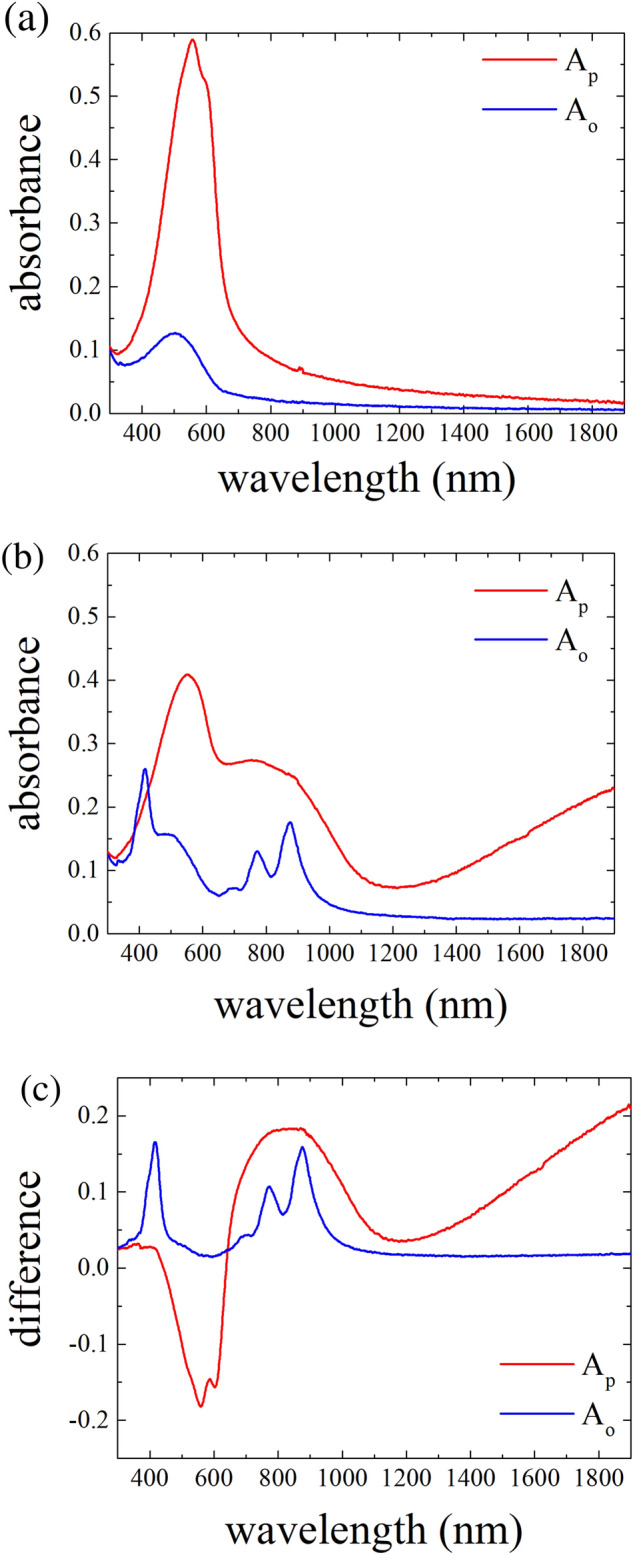
Polarized electronic absorption spectra of ftP3HT film (**a**) before and (**b**) after F4TCNQ-doping. (**c**) Absorption difference of the F4TCNQ-doped ftP3HT film in both polarizations. The directions of polarizations A_p_ (red) and A_o_ (blue) are parallel and orthogonal to the drawing direction of friction-transfer, respectively.

Electronic absorption spectroscopy is widely utilized for the characterization of doped polymeric semiconductors owing to the facile investigation of the subgap optical absorptions from the polaronic species. Polaronic species generated after doping of conjugated polymers were reported to be delocalized in 1D (intrachain) or 2D (interchain) depending on the molecular order present in the system under investigation^3,51,52^. Therefore, polarized absorption spectroscopy conducted on oriented polymeric films and their subsequent doping are expected to provide significant information regarding the nature of the delocalized polaron in such systems. Figure 2(a) shows the polarized UV–vis-NIR absorption spectra of pristine ftP3HT on a glass substrate. The absorption spectrum with parallel polarization (A_p_, red line) exhibits vibronic features from the lamellar structure of P3HT at approximately 560 and 600 nm. By contrast, the absorption spectrum with orthogonal polarization (A_o_, blue line), exhibits a single broad absorption at approximately 500 nm. Pristine ftP3HT displays clear optical anisotropy associated with the polymer backbone alignment because of the π–π* transition moment along with the polymer backbones. The dichroic ratio (D = A_p_/A_o_) was estimated to be 8 at 600 nm and 6 at 560 nm. The P3HT backbones in ftP3HT were highly aligned in the drawing direction of friction transfer^31^. After F4TCNQ-doping by the dipping method, F4TCNQ-doped ftP3HT exhibits unique polarized absorption spectra as shown in Fig. 2(b). The A_p_ absorption spectrum of F4TCNQ-doped ftP3HT exhibits only a typical spectrum according to the P3HT polaron, as indicated by the broad absorption in the NIR region without any spectral feature associated with the F4TCNQ anions. By contrast, the A_o_ absorption spectrum displays only a spectrum according to F4TCNQ anions, indicating sharp peaks appearing at approximately 420, 770 and 870 nm without any absorption owing to the P3HT polaron^17,53^. These findings corroborate that the P3HT polarons are delocalized along the polymer backbones and the F4TCNQ anions are oriented with respect to the aligned P3HT polymeric chains.

Precise analysis of the P3HT-F4TCNQ charge-transfer interactions in the blended solution and the non-oriented doped film requires the deconvolution of the absorption spectra into P3HT polarons and F4TCNQ anions^14,16,18^. In this study, we separately observed the absorption spectra of the P3HT polaron and F4TCNQ anions by simple polarized absorption spectral measurement of F4TCNQ-doped ftP3HT. Figure 2(c) shows the difference in the polarized absorption spectra of ftP3HT before and after F4TCNQ-doping. In the difference spectrum of A_p_ absorption (red line), the absorption band associated with the π–π* transition of pristine P3HT appearing at 524, 558 and 603 nm decreased. Moreover, new broad absorption bands appeared at approximately 758, 927 and above 1300 nm, that can be considered as the three allowed optical transitions, P3, P2, and P1, of the positive polaron in P3HT^18,19,54^. In the difference spectrum of A_o_ absorption (blue line), the sharp absorption of F4TCNQ anions at 402, 418, 700, 771 and 877 nm were seen without any pristine P3HT absorption^55^. These findings corroborate that the direction of the optical transition moment of the F4TCNQ anion aligned orthogonally to that of the P3HT backbone.

### Grazing incidence X-ray diffractometry

To fully investigate the arrangement of P3HT macromolecules in the ftP3HT thin films before and after molecular doping by the F4TCNQ molecules, GIXD measurements were performed. We previously reported the structure of ftP3HT by GIXD and demonstrated that lamellar stacking of P3HT backbones is perpendicular to the drawing direction of friction-transfer and is in the film plane (*q*_*y*_ direction), while the π–π stacking between adjacent polymeric chains in the film is out-of-plane (*q*_*z*_ direction), indicating the 3D alignment of the P3HT macromolecules in ftP3HT thin films^31,33,34^.

**Figure 3:**
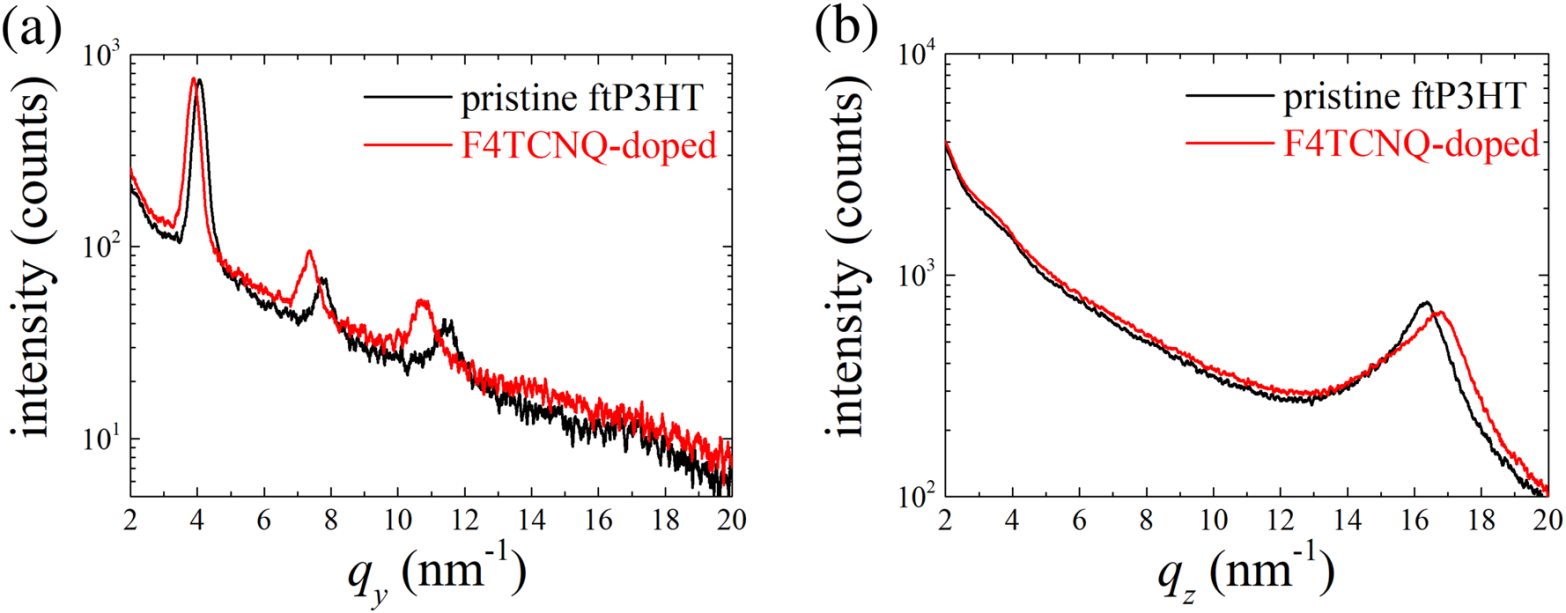
GIXD profiles of F4TCNQ-doped ftP3HT film with (**a**) in-plane and (**b**) out-of-plane geometry.

Figure 3a shows the GIXD profiles in the *q*_*y*_ direction for both the pristine (black line) and F4TCNQ-doped ftP3HT (red line). The profile of the pristine ftP3HT displays periodic scattering peaks according to the (*h*00) reflections corresponding to the lamellar stacking of P3HT backbones. This means that P3HT molecules in ftP3HT were highly aligned in the drawing direction of friction-transfer with laying side-chain in the film plane. The profile of the F4TCNQ-doped ftP3HT also displays periodic (*h*00) reflections with shifts to smaller *q*-values indicating an increase in the lamellar spacing. The lattice strain in ftP3HT was quantified according to the literature procedures^16,56^. It was found that there was 4.64% enhancement in the lattice strain for lamellar stacking after F4TCNQ-doping. This increase in the lamellar spacing is attributed to the intercalation of F4TCNQ molecules to the side-chain region of P3HT. This result is consistent with the results in electrochemically and/or chemically doped P3HT films owing to the the incorporation of anions into the lattice^57^. A lattice strain of 2.63% in the *q*_*y*_ direction was also observed in ftP3HT treated in pure acetonitrile (see Supplementary Information: Fig. S1), indicating that lamellar stacking of P3HT is also affected by swelling caused by the solvent. This implies that the F4TCNQ molecules were able to exchange with solvent molecules (acetonitrile) near the swollen side-chain region in solution^58^.

The GIXD profile in the *q*_*z*_ direction for the pristine and F4TCNQ-doped ftP3HT display a scattering peak according to the (020) reflection corresponding to the π–π stacking between adjacent chains, as shown in Fig. 3(b). Other strong scattering peaks in the *q*_*z*_ profiles were not observed completely. This means that, the P3HT molecules in ftP3HT were structured in the face-on orientation. In contrast to the lamellar stacking expansion, the π–π stacking peak shifted to a larger *q*-value after F4TCNQ-doping in ftP3HT. The π–π stacking after F4TCNQ-doping has a contraction of 2.68% in lattice strain. This contraction was also observed in the electrochemically doped P3HT films^56^. The π–π stacking spacing decreased between adjacent chains owing to the strong inter-chain interactions induced by F4TCNQ-doping. The lattice strain in the *q*_*z*_ direction was not observed in ftP3HT treated in pure acetonitrile (see Supplementary Information: Fig. S1). This means that acetonitrile did not affect the π–π stacking of P3HT.

These GIXD results suggest that F4TCNQ molecules doped in ftP3HT by the dipping method were arranged in the P3HT lamellar spacing of their alkyl side-chains. The solvent effect was also confirmed for ftP3HT following dipping into pure acetonitrile. The lattice strains following dipping into pure acetonitrile were observed to be 2.63% and 0% in the *q*_*y*_ and *q*_*z*_ directions, respectively, indicating that P3HT lamellae were swollen by acetonitrile and that the stacking was not affected by the solvent molecules. Thus, we propose the following mechanistic model for the doping of F4TCNQ into ftP3HT: initially, the solvent (acetonitrile) molecules impart swelling of the P3HT lamellae, followed by their exchange with F4TCNQ molecules by CT interaction between F4TCNQ and P3HT. The side-chain region is important for providing the space of acceptor molecules in this method. Particularly, poly(3-alkylthiophene) s (P3ATs) with other alkyl side-chain lengths, such as butyl, octyl, decyl, and dodecyl, can be doped successfully by the same dipping procedure, although the unsubstituted polythiophene with no side-chains cannot be doped (see Supplementary Information: Fig. S2). Furthermore, strong CT interactions between P3HT and acceptor dopants are important. Because P3HT doping by dipping into TCNQ with no fluorine substituent was not observed, a strong CT interaction between P3HT and the acceptor is necessary for the intercalation of acceptor molecules into the P3HT film.

### Polarized FT-IR spectroscopy

**Figure 4:**
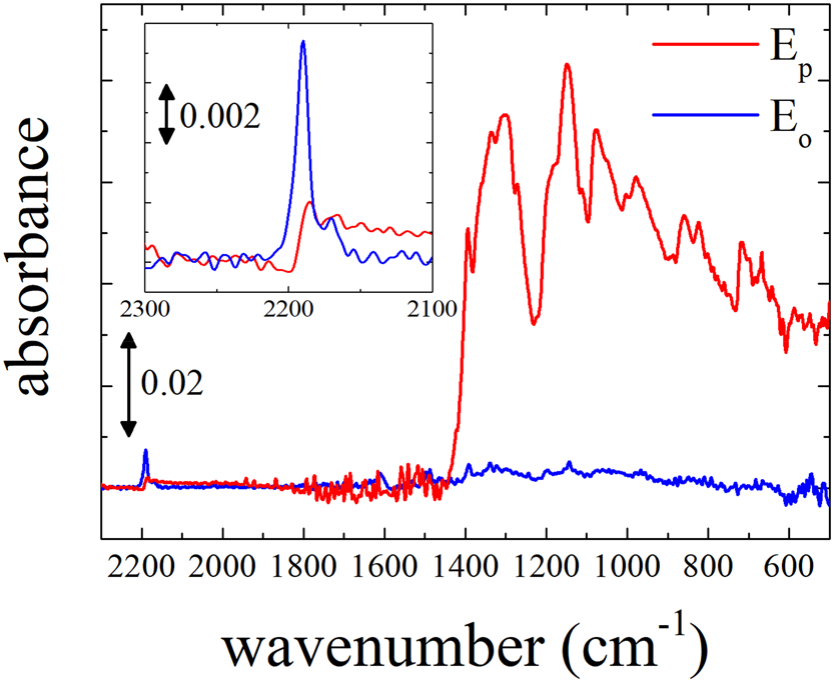
Polarized FT-IR absorption spectra of the F4TCNQ-doped oriented P3HT film. The directions of polarizations E_p_ (red) and E_o_ (blue) are parallel and orthogonal to the drawing direction of friction-transfer, respectively.

The results from the electronic absorption spectroscopy and GIXD investigations corroborate that F4TCNQ anions exist as oriented species in the alky side-chain region in P3HT polymeric chains, and guided by the orientation of the P3HT backbone. From carefully probing of the C≡N stretching vibrations of the F4TCNQ dopant, investigations of the polarized FT-IR and pMAIRS are expected to provide significant information on the molecular orientation of F4TCNQ in this system. Figure 4 shows the polarized FT-IR spectra of the F4TCNQ-doped ftP3HT film prepared on a silicon wafer. The doping-induced modes of P3HT around 1350—500 cm^−1^ are strong in the parallel polarization (E_p_, red line) and are significantly weak or unnoticeable in the orthogonal polarization (E_o_, blue line). This anisotropic feature in the FT-IR spectrum together with the polarized electronic absorption spectra (Fig. 2) presents concrete evidence that P3HT polarons are delocalized along the backbone. The molecular orientation of TCNQ derivatives and their degree of charge-transfer are well-known to be estimated from the wavenumbers of C≡N stretching vibrations in their IR spectra. The magnified section of the spectrum in the wavenumber region around 2200 cm^−1^ associated with the C≡N stretching vibrations is shown in the inset of Fig. 4. The FT-IR spectra corroborates the presence of a strong peak at 2188 and 2170 cm^−1^, which are assigned to the C≡N stretching vibrations from the F4TCNQ anions, whereas peaks associated with the neutral F4TCNQ molecule appearing at 2230and 2220 cm^−1^ were completely absent^14,59^. This indicates that the F4TCNQ molecules in the doped ftP3HT thin film prepared by dipping completely changed to anions. That is, F4TCNQ molecules via dipping diffused into the ftP3HT film by the charge-transfer interactions. This was validated by the experimental evidence that the non-fluorinated analog TCNQ did not exhibit polaronic absorption, if spin coated thin films of P3HT were subjected to solution phase sequentially doping^60^. This is attributed to the lack of integral charge-transfer between the P3HT and TCNQ molecules owing to its lower electron affinity (4.23 eV) compared to that of the ionization energy (4.6 eV) of the polymer^61,62^. Mendez *et al*. also demonstrated that F4TCNQ exhibit charge-transfer interactions with polymeric P3HT and oligomeric analog 4 T, whereas TCNQ does not from experimental and theoretical investigations^14^.

**Figure 5:**
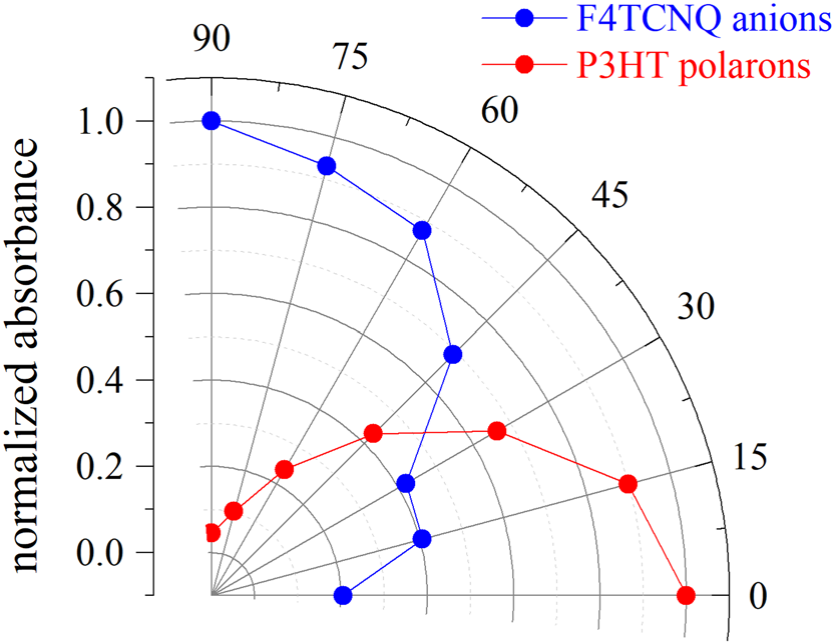
Polar plot of polarized FT-IR absorption at 1150 cm^−1^ on P3HT polarons (red) and at 2188 cm^−1^ on F4TCNQ anions (blue).

Furthermore, the anisotropic feature of the C≡N stretching vibrations owing to F4TCNQ anions was observed in F4TCNQ-doped ftP3HT. The peaks owing to F4TCNQ anions were strong in the E_o_ absorption in contrast to that of P3HT polaron. Figure 5 shows a polar plot (angle-dependent molecular orientation) of the normalized FT-IR absorbance for the two main characteristic vibrations associated with the P3HT polaron at 1150 cm^−1^ and of F4TCNQ anions at 2188 cm^−1.^ This orientation distribution of relative intensities as a function of polarization angle associated with the P3HT polarons further indicated that P3HT backbones are along the drawing direction of friction-transfer. Although the C≡N stretching vibrations of F4TCNQ molecules are in two directions, the orientation distribution of F4TCNQ anions indicates that F4TCNQ molecules are highly aligned with respect to the P3HT backbones.

**Figure 6:**
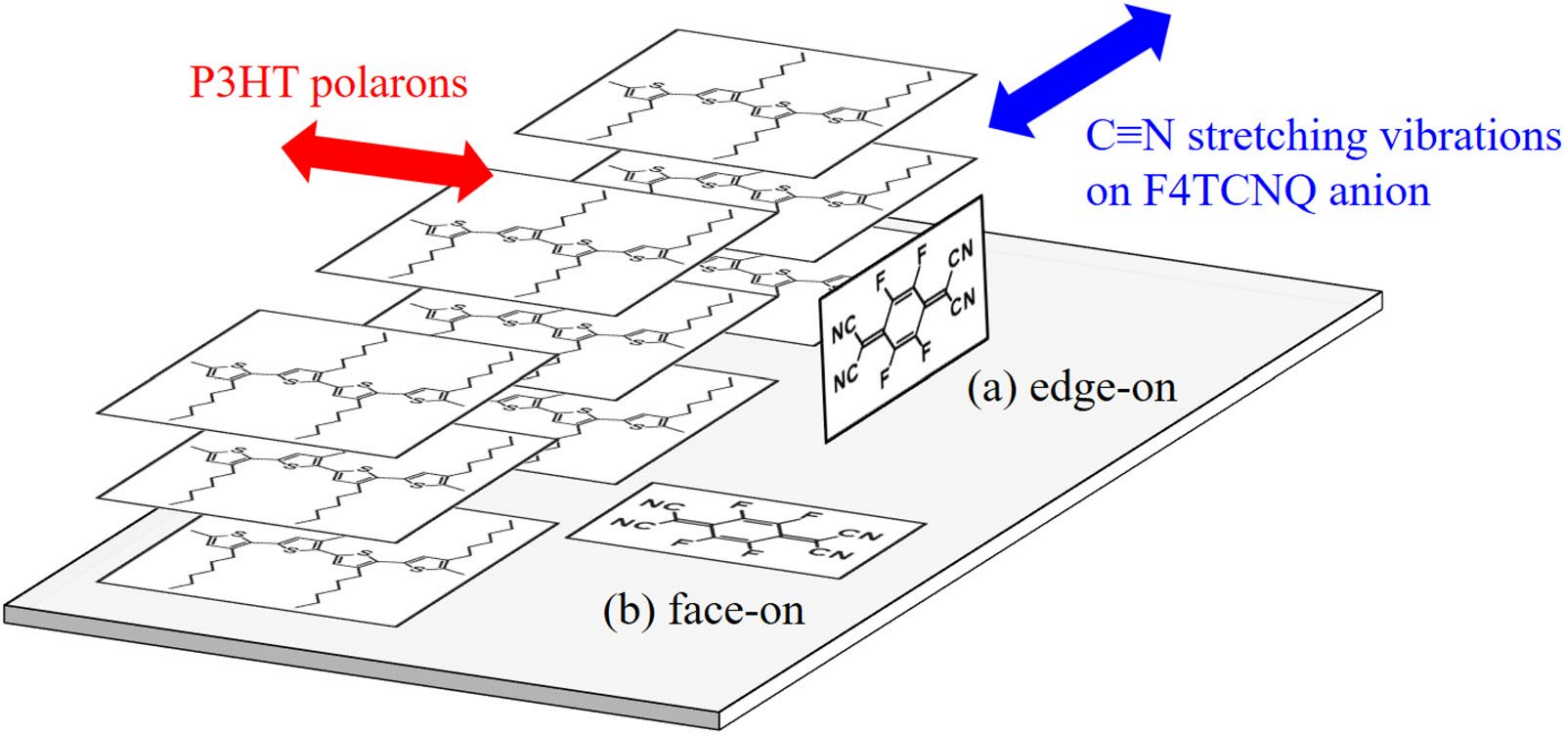
Schematic of the two candidates of the molecular arrangement of F4TCNQ anions in ftP3HT. F4TCNQ anions oriented in the edge-on (**a**) and face-on (**b**).

Two possible models of the molecular arrangement of F4TCNQ anions in ftP3HT are schematically shown in Fig. 6. The edge-on orientation of the F4TCNQ molecular-long-axis perpendicular to the P3HT backbones in shown in Fig. 6(a). This edge-on model was previously reported based on the hypothesis that the direction of the optical transition moment of the F4TCNQ anion is along the molecular long-axis. Without this hypothesis, there is a possibility of face-on orientation of the F4TCNQ molecular long-axis parallel to the P3HT backbones as shown in Fig. 6(b). This is a new finding from this study. This face-on model is consistent with the polarized FT-IR results. This proposal was further validated by probing the orientation of the C≡N stretching vibrations of F4TCNQ in the out-of-plane (a) or in-plane (b) directions.

### pMAIRS spectroscopy

**Figure 7:**
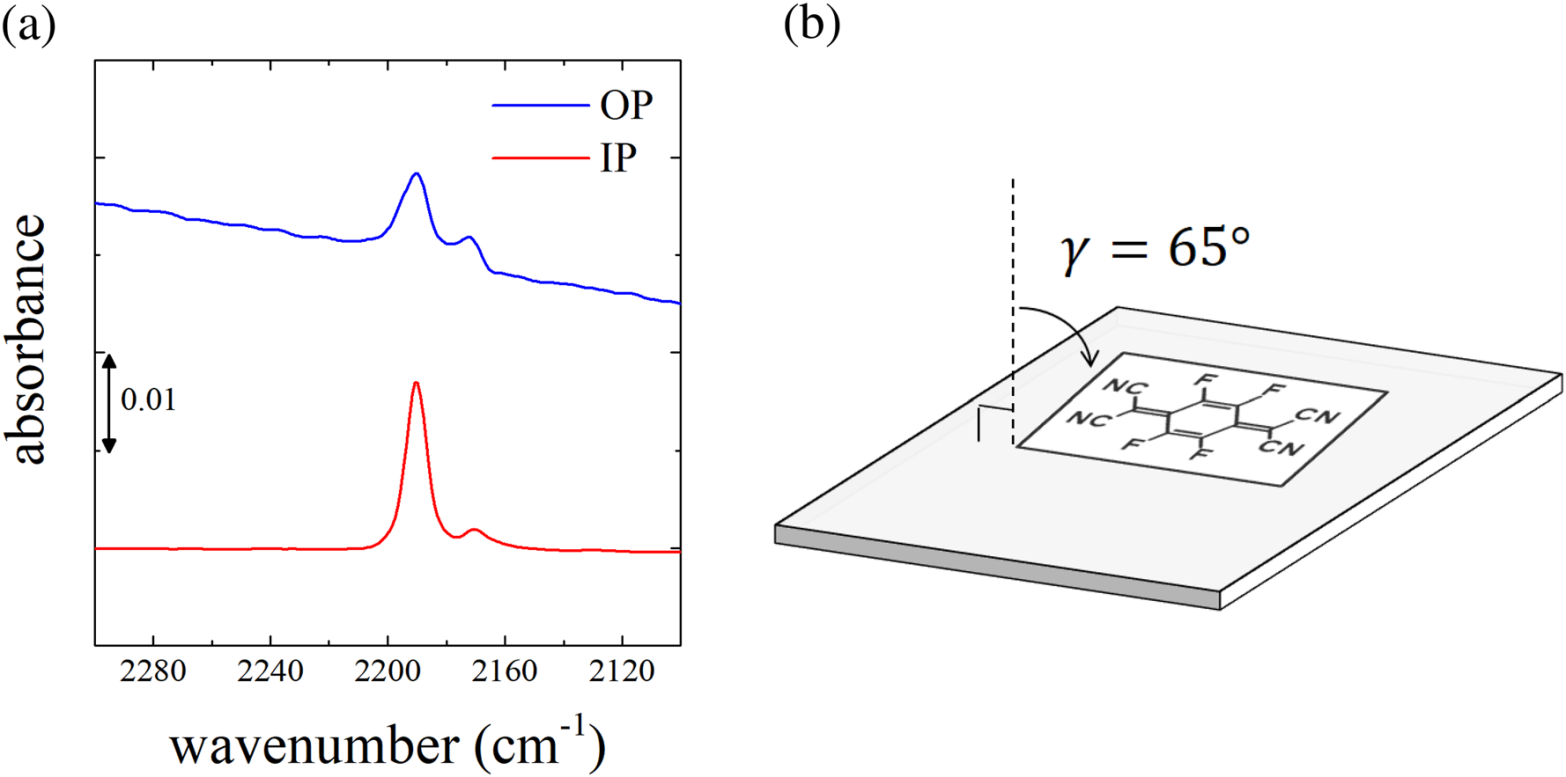
(**a**) pMAIRS spectra of the F4TCNQ-doped ftP3HT film. The red and blue lines correspond to the IP and OP spectra, respectively. (**b**) Schematic of the molecular orientation of F4TCNQ anions.

This is a novel analytical tool to expand the horizon of FT-IR spectroscopy to obtain in-depth knowledge of the molecular orientation by probing the vibration bands under investigation. This technique allows the simultaneous acquisition of the vibrational data for the oriented molecules by extracting only the in-plane and out-of-plane vibration modes from single beam spectra at each incident angle. The pMAIRS spectra provide the orientation angle γ, which is the angle from the surface normal of a focused vibration and is calculated by the dichroic ratio of in-plane (IP, red line) and out-of-plane (OP, blue line)^50^. Figure 7 shows the pMAIRS spectra of F4TCNQ-doped ftP3HT film prepared on silicon wafer focused on the C≡N stretching vibrations of F4TCNQ anions. The C≡N stretching vibrations of F4TCNQ anions are strong in the IP spectra and weak in the OP spectra. The orientation angle γ was calculated to be 65° from the peak intensities (A_IP_ and A_OP_) at 2188 cm^−1^ in the pMAIRS spectra using the equation, γ = tan^−1^(2A_IP_/A_OP_)^1/2^. This indicates that the C≡N stretching vibrations lie almost in the film plane. The pMAIRS analysis results corroborates that F4TCNQ molecules preferentially achieve the face-on orientation in the F4TCNQ-doped ftP3HT film; this is consistent with the schematic of the molecular arrangement shown in Fig. 6(b). Therefore, the direction of the optical transition moments of F4TCNQ anions can be concluded to be along the molecular short-axis.

## Conclusions

We investigated the ordered arrangement of the molecular acceptor, F4TCNQ, in 3D oriented P3HT prepared by the friction-transfer method utilizing polarized UV–vis-NIR, GIXD, polarized FT-IR, and pMAIRS measurements. Acceptor doping was performed by simple dipping of 3D oriented P3HT thin films in an acetonitrile solution of F4TCNQ. The F4TCNQ-doped oriented P3HT showed unique polarized UV–vis-NIR absorption spectral features. Parallel polarization displays only the absorption of P3HT polarons, whereas orthogonal polarization displays only the absorption of F4TCNQ anions. The GIXD profiles showed that the P3HT lamellar spacing expansion and π–π stacking contraction after F4TCNQ-doping. This indicate that the F4TCNQ anions were arranged in the side-chain region of P3HT. Polarized FT-IR absorption showed anisotropies of P3HT polaron-related vibrations and C≡N stretching vibrations of F4TCNQ anions. These results indicate that P3HT polarons were delocalized along the P3HT backbones, wherein F4TCNQ anions align according to the P3HT alignment by charge-transfer interaction. Furthermore, F4TCNQ anions were found to attain a face-on orientation by pMAIRS analysis. The optical investigation results indicated that the direction of the optical transition moment of the F4TCNQ anion is not along the molecular long-axis but along the molecular short-axis. These findings suggest that charge-transfer between P3HT and F4TCNQ may occur without the overlap of each molecular plane and π-electrons. The molecular arrangement of donor and acceptor molecules owing to the charge-transfer interaction can be performed in a microstructural orderly manner, even in intricate polymer systems.

## Methods

### Materials

Regioregular poly(3-hexylthiophene) (P3HT) was purchased from Sigma-Aldrich Co. Ltd. and used after once re-precipitation from the chloroform solution into methanol. The 2,3,5,6-Tetrafuluoro-7,7,8,8-tetracyanoquinodimethane (F4TCNQ) was purchased from Tokyo Chemical Industry Co. Ltd. and used as received. Dehydrated solvents were purchased from Wako Pure Chemical Industry, Ltd. and used as received.

### Film preparation

The 3D oriented P3HT films were prepared on the substrates by the friction-transfer technique, as described in our previous publications^31,32^. For friction-transfer, P3HT powder was compressed into a pellet with an applied load of 1250 kgf cm^-2^ under vacuum. The condition of the friction-transfer procedure was performed by squeezing and drawing a P3HT pellet with a contact area of 0.1 cm^2^ on the heated substrate at a temperature of 100 °C. The applied load for squeezing was 30 kgf cm^-2^, and the drawing speed was 1 m min^−1^. The obtained friction-transferred P3HT films (ftP3HT) had a thickness of approximately 200 nm. Post-doping of F4TCNQ into ftP3HT was performed by the dipping method. The ftP3HT was dipped into F4TCNQ acetonitrile, which is an orthogonal solvent of P3HT, solution with a concentration of 0.1 mg ml^−1^ for 30 min, and thereafter rinsed with pure anhydrous acetonitrile for 30 s followed by substrate drying^28^.

### Optical characterization

The polarized UV–vis-NIR absorption spectrum was measured using a JASCO V-570 spectrophotometer with a Glan–Thompson polarizing prism. The polarized FT-IR spectrum was measured using a JASCO FT/IR-4100 spectrometer with a KRS-5 wire grid polarizer. FT-IR spectra were recorded from 400 to 4000 cm^−1^ with a 4 cm^−1^ resolution and with the integration of 128 scans. Parallel polarization (0°) was defined as along the drawing direction of friction-transfer. Orthogonal polarization (90°) was defined as perpendicular to the drawing direction of friction-transfer. The pMAIRS was measured using a JASCO FT/IR-6700FV spectrometer equipped with a MAIRS attachment^47–50^. The pMAIRS spectra were recorded from 700 to 4000 cm^−1^ with a 4 cm^−1^ resolution with the integration of 200 scans for each incident angles under vacuum condition. The optimal incident angles are employed from 9° to 44° in 5° steps. The pMAIRS was performed at JASCO Corporation.

### Microstructural characterization

GIXD analyses were performed using a Rigaku Smart Lab diffractometer with Cu Kα (λ = 0.154 nm) irradiation. The experimental geometry for this measurement was described in our previous publications^31,33,34^. The grazing angle of the incidence, ω, in this study, was fixed at 0.14°, which is the critical angle for total reflection against the silicon substrate. The oriented polymer films were examined in three principal directions of the scattering vector chosen to relate to the drawing direction of friction-transfer. From these three settings, one can obtain a partial crystal structure of the oriented film^33–36^. The out-of-plane X-ray scattering vector *q*_z_ and the in-plane X-ray scattering vector *q*_xy_ were positioned normal and parallel to the film surface, respectively. The components of the in-plane and out-of-plane X-ray scattering vectors were defined as *q*_xy_ = (4π/λ) sinθχ and *q*_z_ = (2π/λ) (sin(ω) + sin(2θ)), respectively. The angle χ between the in-plane scattering vector and the drawing direction of friction-transfer was fixed during the in-plane φ-2θχ scan. We defined the χ-related scattering vectors *q*_x_ and *q*_y_ for fixed-χ-angles of 0° and 90°, respectively. For the out-of-plane 2θ scan and in-plane φ − 2θχ scans, the scanning speed was 2°/min, and the angular interval between steps was 0.02°.

## Supplementary information

Supplementary Information.
