# Supplementary material for: Ordered arrangement of F4TCNQ anions in three-dimensionally oriented P3HT thin films
Source: Sci Rep. 2020 Nov 18;10:20020. doi: 10.1038/s41598-020-77022-0 (PMC7674482; doi:10.1038/s41598-020-77022-0)
Supplement: Supplementary file 1 — Supplementary Information. [file 41598_2020_77022_MOESM1_ESM.pdf]

# Supplementary Information

# Ordered Arrangement of F4TCNQ Anions in Three-Dimensionally Oriented P3HT Thin Films

Shuichi Nagamatsu[1,*] and Shyam S. Pandey[2]

[1]Department of Physics and Information Technology, Kyushu Institute of Technology, 680-4 Kawazu, Iizuka, Fukuoka, 820-8502, Japan.

[2]Graduate School of Life Science and System Engineering, Kyushu Institute of Technology, 2-4 Hibikino, Wakamatsu-ku, Kitakyushu, 808-0196, Japan.

Correspondence and requests for materials should be addressed to S.N. (email: nagamatu@cse.kyutech.ac.jp)

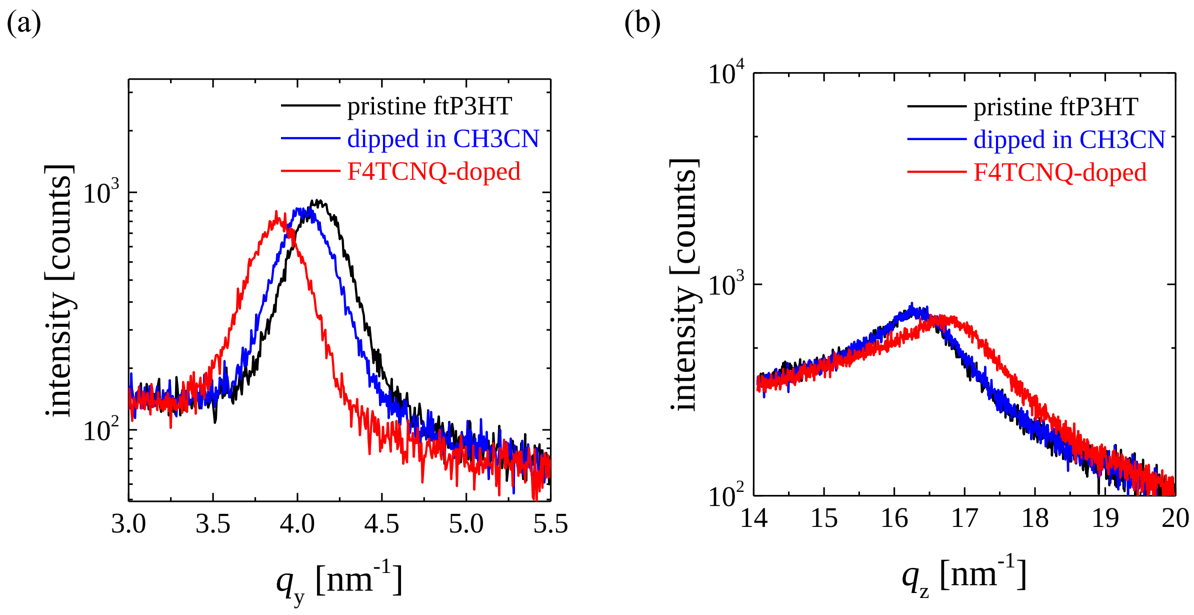


**Figure S1.** GIXD profiles of pristine (black line), dipped in acetonitrile (blue line) and F4TCNQ-doped (red line) ftP3HT film with focusing at (a) in-plane (100) reflection and (b) out-of-plane (020) reflection.

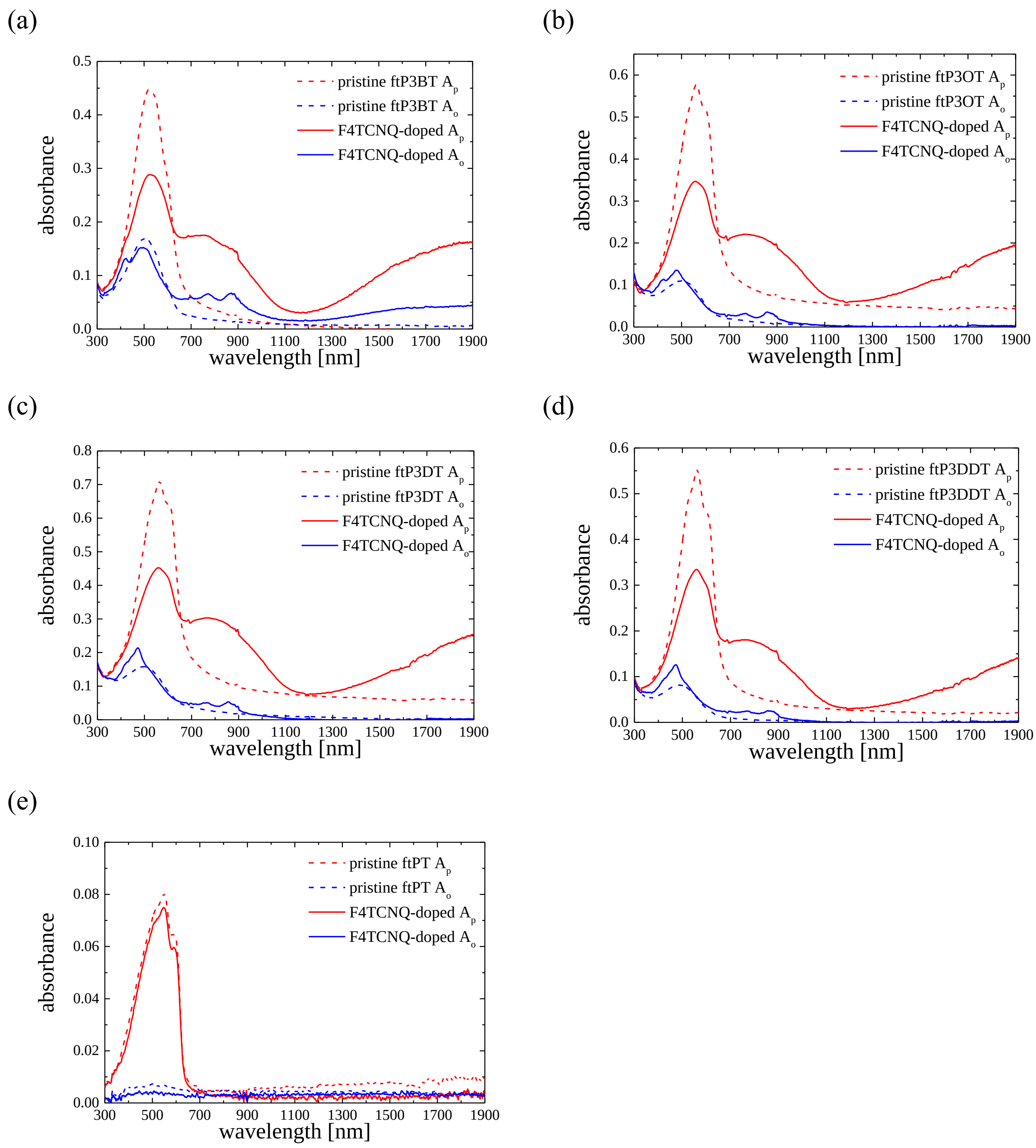


**Figure S2.** Polarized UV-vis-NIR absorption spectra of the friction-transferred polythiophene derivatives films before (dashed line) and after F4TCNQ-doping (solid line). $A_p$ (red) and $A_o$ (blue) polarization are parallel and orthogonal to the drawing direction of friction-transfer, respectively. (a) butyl, (b) octyl, (c) decyl, (d) dodecyl and (e) unsubstituted polythiophene film.

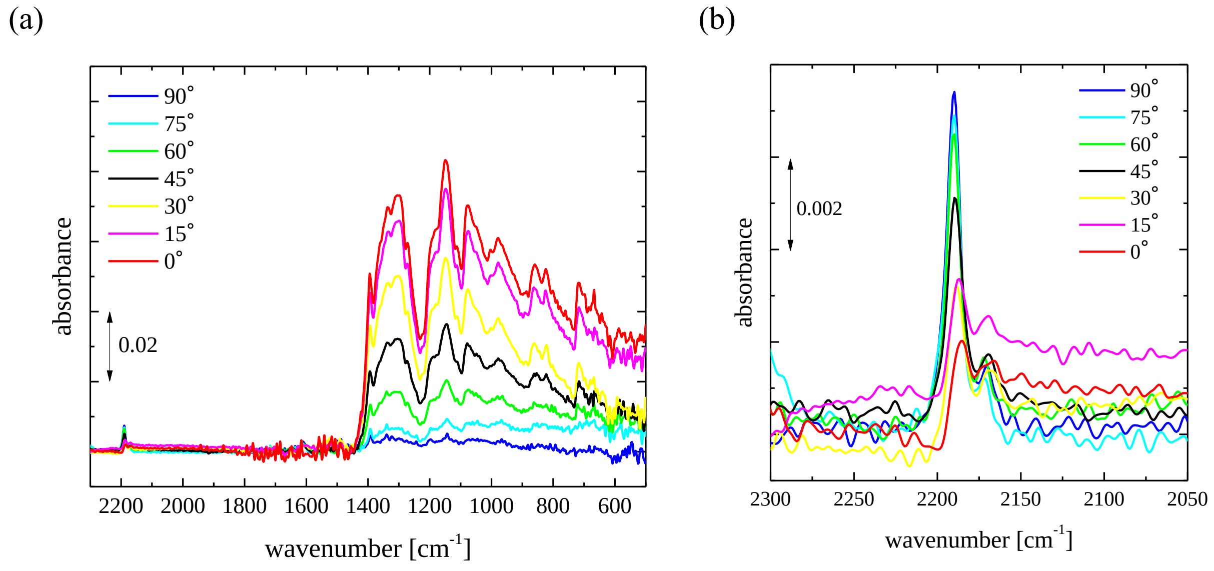


**Figure S3.** Polarized FT-IR spectra of the F4TCNQ doped 3D oriented P3HT film with different polarized-angles.
